# A Pilot Intervention to Promote Positive Parenting in Refugees from Syria in Lebanon and Jordan

**DOI:** 10.3389/fpsyt.2020.00257

**Published:** 2020-04-07

**Authors:** Najla A. Lakkis, Mona H. Osman, Lara C. Aoude, Cosette J. Maalouf, Hanane G. Issa, Ghassan M. Issa

**Affiliations:** ^1^ Department of Family Medicine, American University of Beirut Medical Center (AUB-MC), Beirut, Lebanon; ^2^ Arab Resource Collective, Beirut, Lebanon; ^3^ Faculty of Epidemiology and Population Health, London School of Hygiene & Tropical Medicine, London, United Kingdom

**Keywords:** parenting, early childhood, parent-child interaction, refugees, stress

## Abstract

**Background:**

Early childhood development (ECD) is a crucial milestone that shapes a child’s health, wellbeing, education, and personality. Several factors come into play, and each requires the nurturing care of caregivers. Although the importance of ECD is well understood, the implementation of ECD programs is scarce, especially in poor and vulnerable communities.

**Objective:**

To improve parents’ wellbeing, parenting stress levels, parenting behavior, and discipline strategies after the implementation of a newly designed parenting intervention.

**Participants and Setting:**

Parents from Syria (125 mothers and fathers) in three refugee camps in Lebanon and Jordan.

**Methods:**

This was a pilot cohort study in which parents’ wellbeing, parenting stress levels, parenting behavior, and discipline strategies were evaluated before and after participating in training in the form of interactive and educational sessions to ameliorate their relations and interactions with their children.

**Results:**

By the end of this study, parents’ mental health and wellbeing improved (p < 0.001, Cohen’s d: 0.61) and their parenting index score was reduced (p < 0.001, Cohen’s d: 1.24). Some of their dysfunctional interactions with their children as well as the perceived difficulties and conduct problems in their children aged 3 to 6 years were also reduced significantly.

**Conclusion:**

The intervention used in this study succeeded in improving some aspects of parenting practices and disciplines and in improving the parents’ wellbeing; however, more research is needed to assess its long-term effects on parents and their children. Moreover, some adjustments need to be made in the intervention to be more adapted to the context of refugees and underprivileged communities.

## Background

A child’s brain grows rapidly, mostly in the first 3 to 5 years of life. Throughout this process, neural networks are molded by the interaction among genes, environment, and life experiences i.e. nature and nurture (1, 2).

Early child development (ECD) (i.e. from birth to 8 years) can affect significantly several aspects of the child’s wellbeing, health, competence in literacy and numeracy, criminality, and social and economic participation throughout his/her lifespan (3–5). This period of life is critical to the child’s development and it offers opportunities for positive guidance, healthy growth, and development that lasts a lifetime (6). Nurturing care is crucial for ECD and it includes conditions promoting “health, nutrition, security, safety, responsive caregiving, and opportunities for early learning” (3). It involves “children, their families, and other caregivers, and the places where they interact” (3).

Every child is in need of and has the right to positive responsive and engaged parenting (2, 5). Responsive parenting is defined as the ongoing provision of care and support that a child needs to be able to survive and thrive (6). It includes emotionally supportive, receptive, and mentally stimulating interactions, responsive feeding, and protection from threats (3, 7). Of these interactions, research has highlighted two parenting behaviors that are essential to children’s development (7–14): (1) early and frequent participation in cognitively stimulating activities (like talking, reading, playing, and singing) to enhance a child’s educational trajectory, and (2) sensitive and responsive parent-child interactions during the cognitively stimulating activities and everyday interactions. Research has also revealed that the most crucial chapter of parenting starts early during pregnancy spanning through early childhood (i.e. till the age of 6 years) and it has short and long-term repercussions on children’s bio-psycho-social wellbeing including their emotions, behaviors, education, and cognitive skills (8, 11, 15–17). Despite the solid evidence highlighting the importance of ECD, the implementation of ECD programs (i.e. initiatives that include an explicit focus on responsive caregiving and/or early learning, as defined in the 2017 Lancet series on ECD) is still scarce (18).

Parental wellbeing, family self-sufficiency, and family resilience have been reported to improve family wellbeing (FWB) including developmental parenting and child wellbeing (19). Stress (defined as coping with challenges) was shown to play a critical role in parenting as well. High levels of parenting stress have negative impacts on infants and children’s outcomes (20–22). They are associated with a difficult infant temperament, higher levels of infant negative affectivity, and problems in behaviors of children aged between 3 and 9 years (20, 22). The levels of parenting stress are higher among families from racial and ethnic minority backgrounds (21), which is largely due to higher poverty rates (23).

Parenting practices have a significant influence on children’s development as demonstrated in the literature that studied the links between parenting styles, parental discipline responses, children’s behavior, and children’s psychological wellbeing (24–27). Disciplining children (i.e. the process of teaching them the values and normative behaviors of their society) is one of the most important yet difficult responsibilities of parenting. Parenting programs that improve FWB and reduce parenting stress, promote different positive parenting aspects and disciplines. Such programs are needed worldwide, particularly in poor and marginalized communities.

The objectives of this pilot study are to evaluate parent wellbeing, parenting stress levels, as well as parenting behavior and discipline strategies used by refugees from Syria in bringing up their children in Lebanon and Jordan, before and after the implementation of a parenting intervention that encompasses a structured set of group training sessions and activities for parents in couples. This intervention is intended to positively influence different aspects of parenting such as disciplines and behaviors aiming at achieving positive outcomes for children in the long run. This pilot study will serve as a core element in a long-term project entitled “SANAD” i.e. SUPPORT in Arabic, which aims at training parents in couples to become master trainers and train others in a snowball team building activity.

## Design and Methods

### Study Design and Participants

This pilot cohort study was conducted from June 2017 till April 2018 in three refugee camps in Lebanon (Bourj Brajneh and Shatila camps) and Jordan (Amman camp). The selected camps have been historically home to Palestinian refugees and have since 2011 become the residence of a large number of Syrian and Palestinian refugees from Syria which inflated their overcrowded living conditions. Refugees from Syria living there are generally economically vulnerable and dependent on organizations like the United Nations High Commissioner for Refugees (UNHCR), the World Food Programme (WFP), and other humanitarian organizations. This study is part of the Health, Education and Protection Parenting Project (HEPPP) managed by Arab Resource Collective (ARC) in Lebanon in coordination with Plan International (PI) in Jordan.

The target population included all refugees from Syria who satisfy the following criteria: 1) They have preschoolers (children aged 6 years or younger), 2) they are parents in couples unless unfeasible e.g. traveling partner, widow, divorced, 3) they understand Arabic language, and 4) they can to participate in the complete set of 21 weekly study sessions and discussions, each lasting 2 to 3 h (16 ECD sessions and 5 Mental Health and Psycho-social support sessions) to the best of their capabilities. Parents of children with self-reported psychotic disorder, disruptive behavioral disorder, or substance abuse were excluded from this study.

The directors of community and/or social service centers (identified as community leaders) located within each camp facilitated the recruitment process under the supervision of ARC and PI research coordinators (n = 4). The research coordinators invited the eligible parents to participate in this study after explaining its objectives, phases, and projected benefits and risks (e.g. changing the schedule of sessions which may delay the end of the intervention). They also explained that participation is completely voluntary and that all information exchanged during the interviews will be held in strict confidentiality. Parents who accepted to participate signed an informed consent before the first interview with the coordinators. The recruitment stopped once the required convenient sample size was reached. The participants were only given the transportation fees after every session and were informed that some of them will be selected to become trainers for other parents in the second phase of the program, and will be paid per session.

A total of 125 parents (50 from Bourj Brajneh, 34 from Shatila, and 51 from Amman) were gathered and distributed as 43 couples, 33 mothers, and 6 fathers (i.e. a total of 76 mothers and 49 fathers). Around 53% of the parents (67 parents or 42 mothers and 25 fathers) completed the study (22 from Bourj Brajneh, 12 from Shatila, and 33 from Amman), which is equivalent to 25 couples (58%). Only one father had two wives and all of them were attending the sessions in Shatila camp. The non-couple participants were 19 mothers whose husbands were not in the same country during the intervention period. Dropout was mainly due to a change in life circumstances (e.g. finding a job) or place of living.

### Study Procedures and Instruments

ARC and IC developed a comprehensive intervention in Arabic to positively influence parenting behaviors and achieve favorable outcomes in children. The intervention is composed of the following interactive parenting sessions: an introduction session; pregnancy: towards a healthy beginning; breastfeeding; balanced nutrition system; nutritional problems and indicators; personal hygiene (including proper bath procedures and toilet training); safety and accident prevention; immunity, infection prevention, and diseases; equity and inclusion for all children; communication with and between parents; communication with and between peers; reinforcement of positive behavior; every child has intelligence; what is your child’s?; play: a child’s life; critical thinking, learning, and inquiry-based skills; nursery, kindergarten and school readiness; wrapping up session; in addition to few psycho-social support sessions. The master trainers (1 male and 1 female in each center i.e. a total of 6), psychologists with experience in parenting training and coaching, were responsible for implementing the intervention in a very dynamic way that builds on parents’ current practices and knowledge through interactive activities which include but not limited to: brainstorming activities, group work, roleplay, case study, short presentations, and open focused group discussion.

Before and at the end of the intervention, the research coordinators from ARC and PI conducted a structured interview in Arabic specially designed for this study with the recruited parents. During each interview, which lasted about one hour, the interviewer read aloud each question individually from the printed questionnaire, as some of the participants were illiterate. When any participant expressed confusion about a given question, the interviewer provided only brief explanations or elaborations, typically by paraphrasing the question. The mother and father were interviewed separately in different rooms and/or at different times so that they could not hear each other’s responses. To minimize social desirability, participants were reminded during the interviews that their answers would be kept confidential, that there are no right or wrong answers to the questions, and that it is important for them to answer as honestly as possible.

The structured interview was designed by the research team at ARC based on an earlier research project entitled “Mother-Child Education Program” conducted in partnership with Yale University in the United States (Yale HSC#1403013605). It included parents’ gender, child’s age, and the following instruments: World Health Organization Well-Being 5-item Index (WHO-5) (28–33); Parenting Stress Index-Short Form (PSI-SF) (34, 35); Strengths and Difficulties Questionnaire (SDQ) (36–43), and Parental Discipline Strategies Questionnaire (DSQ) (44) (**Table 1**).

**Table 1:** Description of the Interview Instruments.

Instrument Name	Description
The WHO (five) Well-being Index 1998 version (WHO-5)	A short 5-item self-administered screening instrument that measures psychological well-being including depression among adults in 2–3 min. Used extensively worldwide, translated and validated into different languages, including the Arabic language. Each of the 5 positively worded items is rated on a 6-point Likert scale ranging from 0 (constantly present or all of the time) to 5 (not present or at no time). The summed scores range from 0 to 25. A score < 13 indicates poor wellbeing or low mood, though not necessarily depression and warrants further assessment.
The Parenting Stress Index-Short Form (PSI-SF)	A 36- item self-report questionnaire of parenting stress developed from the full PSI 120-item (duration: 10–15 min). One of the most commonly used measures of parenting stress among parents of children younger than 12 years. It is validated in the Arabic language. Items are rated on a 5-point Likert scale from 1 (strongly disagree) to 5 (strongly agree). It includes four subscales: Defensive Responding (DR), the Parental Distress (PD), Parent-Child Dysfunctional Interaction (PCDI) and Difficult Child (DC) subscales, and a Total Stress scale with internal consistency reliability coefficients of 0.90, 0.89, 0.88, and 0.95 respectively. Higher scores indicate greater levels of parenting stress.
The Strengths and Difficulties Questionnaire (SDQ)	A widely used brief screening questionnaire for psychosocial problems and strengths in the child’s daily life. Appropriate to use in children between 4 and 16 years. The Arabic version of the Children SDQ was validated in the parents and teachers of two samples of Yemeni children. It asks about 25 attributes with three response categories (0 = not true, 1 = somewhat true, and 2 = certainly true), some positive and others negative to cover a broad range of mental health symptoms. It includes 5 impact scales: conduct problems (5 items), hyperactivity-inattention (5 items), emotional symptoms (5 items), and peer relationship problems (5 items), as well as prosocial behaviors (5 items). The first 20 items (i.e. without the prosocial behaviors items) are summed to create a “total difficulty” scale score ranging from 0 to 40, with “high” SDQ scores predicting much greater rates of mental disorders than “low” scores.
Parental Discipline Strategies Questionnaire (DSQ)	It assesses 18 discipline strategies grouped into 7 discipline dimensions: inductive discipline, physical punishment, manipulation of privileges, harsh verbal discipline, argument, shaming, and ignoring. Parents are asked how frequently they use each of the 18 different discipline strategies in the last year on a 5-point scale (1 = never, 2 = less than once a month, 3 = about once a month, 4 = about once a week, 5 = almost every day). The parent’s total score of each discipline dimension is calculated.

References: WHO-5 (28–33), PSI-SF (34, 35), SDQ (36–43), DSQ (44).

### Data Analysis

Data collection and entry were confidential and anonymous. The filled questionnaires were locked in a cabinet that can be accessed by research coordinators only. The Statistical Package for the Social Sciences (SPSS) version 22.0 for Windows (SPSS Inc., Chicago, IL) was used for data analysis. Cronbach’s alpha coefficient (α) was first calculated to assess the internal consistency of each of the selected scales and subscales as a means to evaluate their reliability when utilized as continuous variables. Pre- and post-intervention data were compared using paired-samples t-test in normally distributed data or Wilcoxon signed-rank test in non-normally distributed data. The effect sizes were also calculated using Cohen’s d (d) for paired t-test and r for with Wilcoxon test.

## Results

The mean age of children was 4.0 years at baseline and 4.3 years at the end of the study (16 children were younger than 3 years including 3 *in-utero*, and 49 children were between 3 and 6 years).

The overall instrument reliability was *acceptable for* WHO-5 (α = 0.77), good for PSI-SF and DSQ (α = 0.83 and 0.81 respectively), and questionable for SDQ total difficulties (α = 0.62).

The parents’ WHO-5 improved significantly after the intervention compared to baseline (p <0.001, d: 0.61). This improvement remained significant in parents of children between 3 and 6 years (p < 0.001, d: 0.66), but not for parents of children younger than 3 years (**Table 2**).

**Table 2:** WHO-5 Well-Being Index at baseline and after the intervention.

	Mean (SD)		Paired T-Test			
	Pre	Post	t	df	p-value	Cohen’s d
All Children	10.8 (5.7)	14.3 (5.5)	−4	66	**<.001**	0.61
Children 3 to 6 years	10.9 (5.8)	14.6 (5.4)	−4	49	**<.001**	0.66
Children < 3 years	10.5 (5.8)	13.2 (5.6)	−2	16	0.153	0.46

A p-value less than 0.05 (≤ 0.05) is statistically significant.

The PSI-SF total score and all subscales improved significantly after the intervention compared to baseline (p < 0.001, d: 1.24). This improvement was mostly documented in parents of children between 3 and 6 years (p <0.001, d: 1.31). Only PSI-SF total score, PCDI, and Difficult child subscales improved significantly in parents of children younger than 3 years (p: 0.006, 0.044, and 0.044 respectively; d: 1.07, 0.67, and 0.82 respectively) (**Table 3**).

**Table 3:** PSI-SF, SDQ, and DSQ scales and subscales at baseline and after the intervention.

			Mean (SD)		Paired T-Test				Wilcoxon Test		
			Pre	Post	T	df	p-value	d	Z	p-value	r
**PSI-SF**	All Children	Defensive Responding	18.9 (2.8)	16.6 (2.7)	5.431	66	**<.001**	0.85			
		Parental Distress	31.9 (4.5)	27.8 (4.2)					−5.362	**<.001**	0.46
		Parent-Child Dysfunctional Interaction	28.4 (4.0)	24.9 (3.3)					−4.645	**<.001**	0.41
		Difficult Child	30.7 (4.6)	26.7 (4.0)	5.459	65	**<.001**	0.92			
		Total score	91.1 (9.6)	79.5 (9.0)	7.625	65	**<.001**	1.24			
	Children 3 to 6 yrs	Defensive Responding	19.0 (2.9)	16.4 (2.5)	5.461	49	**<.001**	0.98			
		Parental Distress	32.2 (4.8)	27.4 (3.9)					−5.171	**<.001**	0.52
		Parent-Child Dysfunctional Interaction	28.6 (4.3)	24.6 (3.4)	5.053	48	**<.001**	1.03			
		Difficult Child	30.7 (5.1)	26.4 (4.0)	5.015	48	**<.001**	0.95			
		Total score	91.8 (10.7)	78.5 (9.4)	7.114	48	**<.001**	1.31			
	Children < 3 yrs	Defensive Responding	18.6 (2.3)	17.4 (3.2)	1.519	16	.148	0.46			
		Parental Distress	31.1 (3.5)	28.9 (5.2)					−1.354	.176	0.23
		Parent-Child Dysfunctional Interaction	27.6 (2.6)	25.8 (3.0)	2.186	16	**.044**	0.67			
		Difficult Child	30.4 (2.6)	27.6 (4.0)	2.186	16	**.044**	0.82			
		Total score	89.2 (5.4)	82.4 (7.2)	3.198	16	**.006**	1.07			
**SDQ**	Children 3 to 6 yrs	Emotional Problems Scale	4.6 (2.3)	3.8 (3.1)					−1.181	.238	0.12
		Conduct Problems Scale	3.3 (2.0)	2.2 (2.1)					−2.951	**.003**	0.30
		Hyperactive Scale	4.9 (2.4)	3.9 (2.4)	2.607	48	**.012**	0.42			
		Peer Problems Scale	2.9 (1.7)	3.3 (2.3)					−0.837	.403	0.08
		Prosocial Scale	7.9 (2.0)	7.9 (2.8)					−0.929	.353	0.09
		Total Difficulties Score	15.7 (4.8)	13.2 (7.2)	2.286	48	**.027**	0.40			
**DSQ**	Children 3 to 6 yrs	Inductive Discipline score	8.2 (1.6)	7.6 (1.9)					−1.694	.090	0.17
		Manipulating Privileges score	12.9 (3.0)	10.3 (3.8)	4.176	46	**< .001**	0.75			
		Physical Punishment score	6.6 (2.8)	5.7 (2.8)					−2.089	**.037**	0.21
		Argument score	10.7 (2.9)	9.9 (3.5)					−1.953	.050	0.20
		Harsh Verbal Discipline score	6.5 (2.8)	6.1 (2.9)					−1.250	.211	0.13
		Shaming score	4.6 (2.4)	3.6 (2.3)					−2.172	**.030**	0.22
		Ignoring score	3.2 (1.4)	2.3 (1.3)					−3.162	**.002**	0.32

PSI-SF, Parenting Stress Index-Short Form; SDQ, Strengths & Difficulties Questionnaire; DSQ, Disciplinary Style Questionnaire.

d = Cohen’s d (effect size for paired t test); r (effect size for Wilcoxon test).

A p-value less than 0.05 (≤ 0.05) is statistically significant.

The SDQ total difficulties, as well as the conduct problems and the hyperactive scales, improved significantly after the intervention compared to baseline in parents of children between 3 and 6 years (p <0.027, 0.03 and 0.012 respectively) with moderate effect sizes (**Table 3**).

As for the Parental DSQ, the ignoring, shaming, and physical punishment scores improved significantly in parents of children between 3 and 6 years (p: 0.02, 0.30, and 0.37 respectively) with small effect sizes. There was also a trend towards improvement in the argument score in parents of children between 3 and 6 years (p: 0.050) with small effect size. A significant decline in the manipulating privileges score was found in parents of children between 3 and 6 years (p < 0.001) with moderate to size effect size (**Table 3**).

Cronbach’s alpha coefficient (α) was poor to questionable for the SDQ & DSQ subscales, so the results per item were reported for the SDQ & DSQ in parents of children between 3 and 6 years in the **Tables 4** and **5** respectively.

**Table 4:** Strengths & Difficulties Questionnaire (SDQ).

	Children 3 to 6 years			
	Median (SD)		Wilcoxon Test	
	Pre	Post	Z	p-value
Emotional problems				
Often complains of headaches	1.0 (0.85)	0 (0.91)	−.129	.897
Many worries	1.0 (0.75)	0 (0.94)	−1.143	.253
Often unhappy, downhearted	1.0 (0.84)	1.0 (0.87)	−.749	.454
Nervous or clingy in new situations	1.0 (0.80)	0 (0.91)	−2.663	**.008**
Many fears, easily scared	1.0 (0.82)	0 (0.85)	−2.649	**.008**
Conduct Problems				
Often has temper tantrums or hot tempers	1.0 (0.85)	0 (0.80)	−2.539	**.011**
Generally obedient	0 (0.71)	0 (0.77)	−.203	.839
Often fights with other children	1.0 (0.85)	0 (0.88)	−1.540	.124
Often lies or cheats	0 (0.71)	0 (0.52)	−1.618	.106
Steals from home, school, or elsewhere	0 (0.66)	0 (0.46)	−1.748	.080
Hyperactivity				
Restless, overactive	2.0 (0.77)	1.0 (0.88)	−2.737	**.006**
Constantly fidgeting or squirming	1.0 (0.79)	0 (0.81)	−1.394	.163
Easily distracted, concentration wanders	1.0 (0.84)	0 (0.81)	−**2.011**	**.044**
Thinks things out before acting	1.0 (0.89)	1.0 (0.79)	−.557	.577
Sees tasks through to the end	1.0 (0.78)	0 (0.76)	−.841	.400
Peer Problems				
Rather solitary, tends to play alone	0 (0.73)	0 (0.89)	−1.253	.210
Has at least one good friend	0 (0.77)	0 (0.82)	−.557	.578
Generally liked by other children	0 (0.60)	0 (0.69)	−.789	.430
Picked on or bullied	0 (0.83)	0 (0.87)	−.707	.480
Gets on better with adults than with other children	1.0 (0.82)	2.0 (0.93)	−.816	.414
Prosocial				
Considerate of other people’s feelings	2.0 (0.67)	2.0 (0.71)	−.269	.788
Shares readily with other children	2.0 (0.58)	2.0 (0.94)	−2.804	**.005**
Helpful if someone is hurt	2.0 (0.68)	2.0 (0.67)	−.362	.717
Kind to younger children	2.0 (0.82)	2.0 (0.72)	−.886	.375
Often volunteers to help others	2.0 (0.71)	2.0 (0.58)	−1.116	.264

A p-value less than 0.05 (≤ 0.05) is statistically significant.

**Table 5:** Disciplinary Style Questionnaire (DSQ).

How frequently do you …	Children 3 to 6 years			
	Median (SD)		Wilcoxon Test	
	Pre	Post	Z	p-value
Inductive Disciplines				
teach your child about good & bad behavior? Like it’s not nice to hit, or it’s polite to say thank you.	5.0 (0.83)	4.0 (0.98)	−1.454	.146
get your child to apologize or make amends?	4.0 (1.26)	4.0 (1.22)	−1.726	.084
Manipulating Privileges				
give your child a time out or send him/her to his/her room?	3.0 (1.54)	2.0 (1.41)	−1.586	.113
take away privileges? (e.g., no TV after dinner because of misbehavior)	3.0 (1.47)	1.0 (1.21)	−2.964	**.003**
give your child extra chores?	3.5 (1.55)	2.0 (1.71)	−1.883	.060
promise a treat or privilege to your child for good behavior?	4.0 (1.04)	4.0 (1.67)	−2.733	**.006**
Physical Punishment				
spank, slap, or hit your child?	2.0 (1.44)	1.0 (1.33)	−3.203	**.001**
grab or shake your child?	2.0 (1.49)	1.0 (1.47)	−1.227	.220
throw something at your child?	1.0 (1.14)	1.0 (1.33)	−0.833	.405
Argument				
argue with your child?	4.0 (1.38)	4.0 (1.65)	−1.722	.085
yell or shout at your child?	4.0 (1.12)	4.0 (1.53)	−2.067	**.039**
threaten your child with some punishment?	4.0 (1.46)	4.0 (1.42)	−0.652	.514
Harsh Verbal Discipline				
tell your child you won’t love him/her if she/he acts that way again?	2.0 (1.42)	2.0 (1.65)	−1.163	.245
threaten to leave your child?	1.0 (1.15)	1.0 (1.06)	−1.174	.240
try to scare your child into behaving (e.g., by threatening to call the police, tell the teacher, etc.)?	2.0 (1.55)	1.0 (1.19)	−1.678	.093
Shaming				
say you are disappointed with your child or say that his/her misbehavior hurt your feelings?	2.0 (1.41)	1.0 (1.21)	−2.297	**.022**
tell your child s/he should be ashamed of her/himself?	2.0 (1.47)	1.0 (1.40)	−1.302	.193
Ignoring				
ignore your child?	4.0 (1.43)	2.0 (1.31)	−3.162	**.002**

A p-value less than 0.05 (≤ 0.05) is statistically significant.

## Discussion

Parenting practices are significant modifiable aspects of a child’s home environment, which can be targeted to enhance early child development (8, 45). Our intervention to enhance parenting methods among refugees from Syria is the first one conducted among refugees and including couples or fathers.

As the proverb says “you cannot give what you don’t have”; hence, parents’ psychological wellbeing is a critical foundation to positive parenting and children’s psychological wellbeing (19, 45). Mental health difficulties can negatively affect parenting, family functioning, and children’s wellbeing and development (46, 47). Studies have shown also that high parenting stress has negative impacts on infants and children’s outcomes (20–22, 46). This intervention improved the wellbeing of the participating refugee parents, decreased their distress, defensive responding, and dysfunctional interaction with their children, as well as their perception of their children as being difficult children. The effectiveness of group parenting programs in improving maternal psychosocial health in the short-term, including reducing stress, anxiety, and depression was reported in the literature (46). A Cochrane review showed that group-based parenting programs improve parent-child interaction in terms of a reduction in negative behaviors, and an increase in positive behaviors (46). However, these studies were conducted in developed countries and not in environments of human insecurity and displacement.

This intervention held by ARC improved the SDQ total difficulties as well as the conduct problems and the hyperactive scales of children aged 3 to 6 years as rated by their parents post-intervention. In parallel to these findings, a Cochrane review presented some evidence for the use of group-based parenting programs to improve the overall emotional and behavioral adjustment of children with a maximum mean age of 3 years and 11 months, in the short-term (46).

Positive discipline styles, characterized by the presence of warm parents’ behavior, provision of reasoning and explanations to children and teaching them the right behaviors, were found to be more effective in promoting strong parent-child relationship and better overall child wellbeing than negative styles characterized by punitive and power-focused behavior (19). Studies revealed also that low socioeconomic status has been associated with greater levels of harsh, physical, and inconsistent discipline (48), less responsiveness (49), and less supportive and involved parenting (50, 51). A group parenting intervention for Latino families minimized parents’ harsh/inconsistent discipline but did not have a significant effect on their appropriate/positive discipline (52). Different systematic reviews and meta-analyses showed that parenting programs (including home-visiting programs) reduce child maltreatment as well as harsh and dysfunctional parenting practices in the short-term (53–55). Also, a brief intervention with or without a primary care visit can affect parents’ attitudes by decreasing physical punishment in the short-term (56, 57), and improving positive parenting as well as parent-child interaction (8). Our intervention reduced mostly the ignoring, shaming, and physical punishment scores in parents of children between 3 and 6 years, with a borderline decline in the argument score. However, manipulating privileges score deteriorated after the intervention. This can be explained by the fact that the refugees have been struggling to survive since they left Syria to Lebanon and Jordan. They have had difficulties in keeping up with basic needs (shelters and food), therefore they do not possess the luxury to positively reinforce (e.g., promise a treat or privilege to the child for good behavior) or negatively punish their children (e.g., take away privileges such as watching television after dinner because of misbehavior). The “Manipulating privileges” items should be amended to fit the life and abilities of underprivileged refugees.

### Limitations

This is a pilot intervention, and the generalizability of its results is limited because some parents did not choose or were unable to participate in the parent-training intervention as offered. Socioeconomic variables were not collected. The effects of the intervention on the children were only measured indirectly through the subjective reports of the parents. Finally, it is difficult to determine whether changes are a result of the passing of time, or the intervention, as there is no control group.

## Conclusion

Children exposed to war, forced migration, and poor living conditions are vulnerable to long-lasting negative influences and experiences that hinder proper growth and early childhood development. Nurturing care is crucial to create a protective and responsive environment, helping children to prosper even in crisis settings.

In this pilot study, mothers and fathers in three refugee camps in Lebanon and Jordan were trained through interactive sessions that aimed at fostering their relations and interactions with their children. By the end of the program, parents experienced less distress, less defensive responding and dysfunctional parenting, and less perceived difficulties and conduct problems in children. Moreover, their mental health and wellbeing improved in the short term. However, some adjustments need to be made to the intervention tools to be better adapted to refugee and underprivileged settings. Wrap up sessions and financial incentives could decrease the rate of dropout rate. However. this could not be done because of the limited budget. More research is needed as well to assess the long-term effects of such interventions on early childhood development in humanitarian settings.

## Data Availability Statement

The datasets generated for this study are available on request to the corresponding author.

## Ethics Statement

The studies involving human participants were reviewed and approved by the Institutional Review Board (IRB) at the American University of Beirut. The board's approval was secured before using the de-identified data for publication purposes. Parents who accepted to participate signed an informed consent form before the first interview with the coordinators.

## Author Contributions:

GI, LA, and CM designed the intervention, developed the intervention materials, and coordinated its implementation. NL analyzed and interpreted the data. NL, MO, and HI wrote the manuscript. All authors read and approved the final manuscript.

## Funding

This work was supported by the Early Childhood Program, Open Society Foundation, London. The funding source did not contribute to the design of the intervention, the data collection, analysis, or write-up of the results.

## Conflict of Interest

The authors declare that the research was conducted in the absence of any commercial or financial relationships that could be construed as a potential conflict of interest.
